# Risk of Gastrointestinal Bleeding in Patients with End-Stage Renal Disease: The Link between Gut, Heart, and Kidneys

**DOI:** 10.1155/2023/9986157

**Published:** 2023-05-08

**Authors:** Avleen Kaur, Syed M. Baqir, Kundan Jana, Kalyana C. Janga

**Affiliations:** ^1^Department of Medicine, Maimonides Medical Center, Brooklyn, NY 11219, USA; ^2^Department of Nephrology, Maimonides Medical Center, Brooklyn, NY 11219, USA

## Abstract

Patients with end-stage renal disease (ESRD) have a five times higher risk of gastrointestinal bleed (GIB) and mortality than the general population. Aortic stenosis (AS) has been associated with GIB from intestinal angiodysplasia. In this retrospective analysis, we obtained data from the 2012 and 2019 National Inpatient Sample. The primary outcome of interest was all-cause in-hospital mortality and risk factors of mortality in patients with ESRD with GIB with aortic valve disorders especially AS. We identified all patients (≥18 years of age) with ESRD (*n* = 1,707,452) and analyzed based on discharge diagnosis of valvular heart disease (*n* = 6521) in patients with GIB compared with those without GIB (*n* = 116,560). Survey statistical methods accounting for strata and weighted data were used for analysis using survey packages in R (version 4.0). Baseline categorical data were compared using Rao-Scott chi square test, and continuous data were compared using Student's *t*-test. Covariates were assessed using univariate regression analysis, and factors with *p* value less than 0.1 in the univariate analysis were entered in the final model. The univariate and multivariable associations of presumed risk factors of mortality in ESRD with GIB patients were performed by Cox proportional hazards model censored at length of stay. Propensity score matching was done using MatchIt package in R (version 4.3.0). 1 : 1 nearest neighbour matching was done with propensity scores estimated through logistic regression, in which occurrence of GIB, valvular lesions, and AS was regressed according to other patient characteristics. Among patients with ESRD with valvular heart diseases, AS was found to be associated with increased risk of GIB (adj.OR = 1.005; 95% CI 1.003–1.008; *p* < 0.01). ESRD patients with AS showed increased risk of lower GIB (OR = 1.04; 95% CI 1.01–1.06; *p* = 0.02), colonic angiodysplasia (OR = 1.03; 95% CI 1.01–1.05; *p* < 0.01), stomach and duodenal angiodysplasia (OR = 1.03; 95% CI 1.02–1.06; *p* < 0.01), need for blood transfusion add pressors as compared to those without AS. However, there was no increased risk of mortality (OR = 0.97; 95% CI 0.95–0.99; *p* < 0.01).

## 1. Introduction

Gastrointestinal bleeding (GIB) accounts for over half a million admissions annually in the United States [1]. Patients with end-stage renal disease (ESRD) have a five times higher risk of GIB and mortality than the general population [2, 3]. An incidence of GIB of 161 per 1000 person-years has been reported from initiation of hemodialysis (HD) [4]. Angiodysplasia is acquired from small lesions (generally <5 mm). The etiology of angiodysplasia is multifactorial ranging from age-related degeneration of connective tissue and decreased gastric mucosal perfusion in aortic stenosis (AS), and inhibition of von Willebrand factor (vWF) leads to increased angiogenesis [5].

Previous studies have shown that angiodysplasias are the most common cause of GIB in HD patients and that 47% of Chronic Kidney Disease (CKD) patients had angiodysplasias compared with 17% in controls [6]. Similarly, cardiac valvular lesions have been implicated in increased risk of GIB from using antithrombotic medications like aspirin, antiplatelets, anticoagulants, and nonsteroidal anti-inflammatory drugs [7]. Moreover, AS has also been reported to be associated with GIB from intestinal angiodysplasia and has been termed Heyde's syndrome (HS).

In the present literature, the risk and mortality of GIB in patients with cardiac valvular lesions and ESRD have been explored separately; however, their concurrent effect remains uninvestigated. To our knowledge, this is the first study to evaluate whether the concurrence of ESRD and valvular lesions leads to increased mortality. We additionally aim to assess morbidity and predictors of mortality in the above-mentioned patient population.

## 2. Materials and Methods

### 2.1. Study Data

In this retrospective analysis, we obtained data from the 2012 and 2019 National Inpatient Sample (NIS), sponsored by the Agency for Healthcare Research and Quality, as a part of the Healthcare Cost and Utilization Project.

### 2.2. Study Design

Patients' identifications are not revealed in these data. Thus, an institutional review board approval was not required for this study. We identified all patients (≥18 years of age) who had a discharge diagnosis of ESRD (*n* = 1,707,452) using their respective ICD-9/CM and ICD-10-CM codes. Among the ESRD patients, we excluded the ones without GIB. All patients with upper or lower GIB were identified based on ICD-9/ICD-10 codes. We excluded patients with age <18 years. For baseline characteristics, we used patient demographics (age, race, and sex), relevant comorbidities: coronary artery disease (CAD) or CAD equivalent, peripheral vascular disease (PVD), stroke, liver disease, malignancy, hypertension (HTN), diabetes mellitus (DM), chronic lung disease, tobacco smoking, alcohol use, blood thinner use (anticoagulants/antithrombotic/antiplatelets), nonsteroidal anti-inflammatory drug use, and congestive heart failure (CHF; Table 1). We further analyzed patients with ESRD with GIB for outcomes and risk factors. We additionally analyzed patients with ESRD with GIB based on discharge diagnosis of presence (*n* = 6521) or absence (*n* = 75,802) of valvular heart disease focusing on aortic valve disorders, especially AS.

### 2.3. Statistical Analysis

Survey statistical methods accounting for strata and weighted data were used for analysis using survey packages in R (version 4.0). Categorical variables are summarized as percentages, and continuous variables are presented as mean ± SD. Baseline categorical data were compared using Rao-Scott chi square test, and continuous data were compared using Student's *t*-test. Identification of risk factors was done using logistic regression models with inverse probability weighing for complex survey data. Covariates were assessed using univariate regression analysis, and factors with *p* value less than 0.1 in the univariate analysis were entered in the final model. The univariate and multivariable associations of presumed risk factors of mortality in ESRD with GIB patients were performed by Cox proportional hazards model censored at length of stay (Supplementary Table 1). Propensity score matching was done using MatchIt package in R (version 4.3.0). 1 : 1 nearest neighbour matching was done with propensity scores estimated through logistic regression, in which occurrence of GIB, valvular lesions and AS were regressed according to other patient characteristics. Subsequently, outcomes like mortality, mechanical ventilation, need for pressors, need for blood transfusion, and occurrence of angiodysplasias among others were studied in the matched model using logistic regression. Data extraction with ICD-9 and ICD-10 codes was done using Python (version 3.9.1). Statistical analysis was carried out using R (version 3.6.2 R Foundation for Statistical Computing, Vienna, Austria).

### 2.4. Outcomes

The primary outcome of interest was all-cause in-hospital mortality and risk factors of mortality in patients with ESRD with GIB with aortic valve disorders especially AS.

The secondary outcomes included the length, shock requiring vasopressors, acute respiratory failure, and mechanical ventilation. Complications were identified using their respective ICD-9-CM and ICD-10-CM codes.

## 3. Results

**Figure 1:**
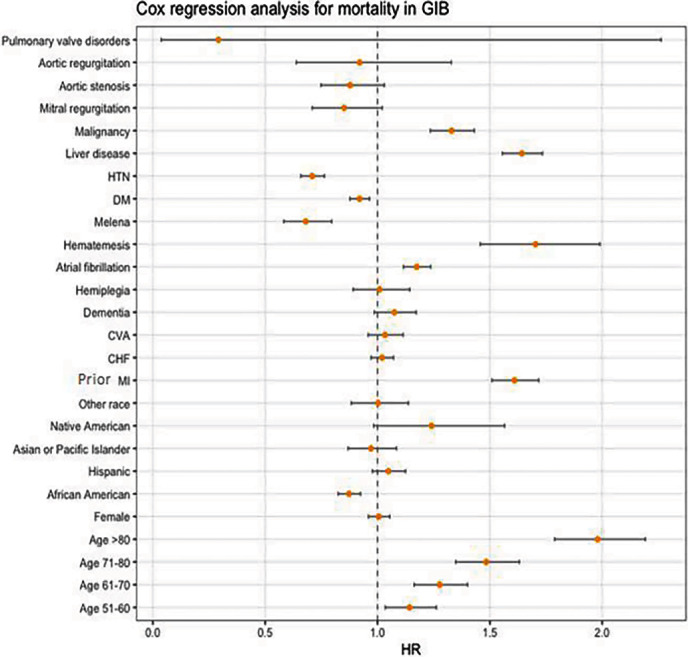
Cox regression analysis for mortality in GIB. HTN: hypertension; DM: diabetes mellitus; CVA: cerebrovascular disease; CHF: congestive heart failure; MI: myocardial infarction.

A total of 1,707,452 patients with ESRD were analyzed. Out of this total, 532,061 patients were on HD. Of the total number of patients, 82,330 patients were diagnosed with GIB over the course of their hospitalization. Comparison of baseline characteristics of ESRD patients with and without GIB is summarized in Table 1. Mean age of patients admitted with GIB was significantly higher than those without GIB (*p* < 0.01). Length of stay, mortality, need for mechanical ventilation, and extracorporeal membrane oxygenation (ECMO) were also significantly higher in patients with GIB. A propensity match analysis between the groups (Supplementary Table 2; Figure 1) also showed a significantly higher risk of mortality (OR = 1.034; 95% CI 1.031–1.036; *p* < 0.01), need for mechanical ventilation (OR = 1.069; 95% CI 1.065–1.073; *p* < 0.01), need for pressors (OR = 1.006; 95% CI 1.003–1.008; *p* < 0.01), and increased length of stay (6.92 ± 9.01 vs. 9.82 ± 14.5; *p* < 0.01; Supplementary Table 3).

Among ESRD patients with GIB, while increasing age was associated with higher mortality, gender and race did not seem to have any associated risks (Supplementary Table 2). Comorbidities like prior myocardial infarction (MI; adj.HR = 1.61; 95% CI 1.51–1.72; *p* < 0.01), atrial fibrillation (adj.HR = 1.18; 95% CI 1.12–1.24; *p* < 0.01), chronic liver disease (adj.HR = 1.64; 95% CI 1.56–1.73; *p* < 0.01), and malignancy (adj.HR = 1.33; 95% CI 1.24–1.43; *p* < 0.01) showed increased risk of mortality, whereas presence of valvular heart diseases, DM, and HTN was not associated with increased risk (Supplementary Table 2). Interestingly, patients presenting with hematemesis had a 1.5 times higher risk of mortality than those presenting with melena (adj.HR = 1.7 vs. adj.HR = 0.68; 95% CI 1.46–1.99 vs. 95% CI 0.58–0.8; *p* < 0.01; Supplementary Table 2).

**Figure 2:**
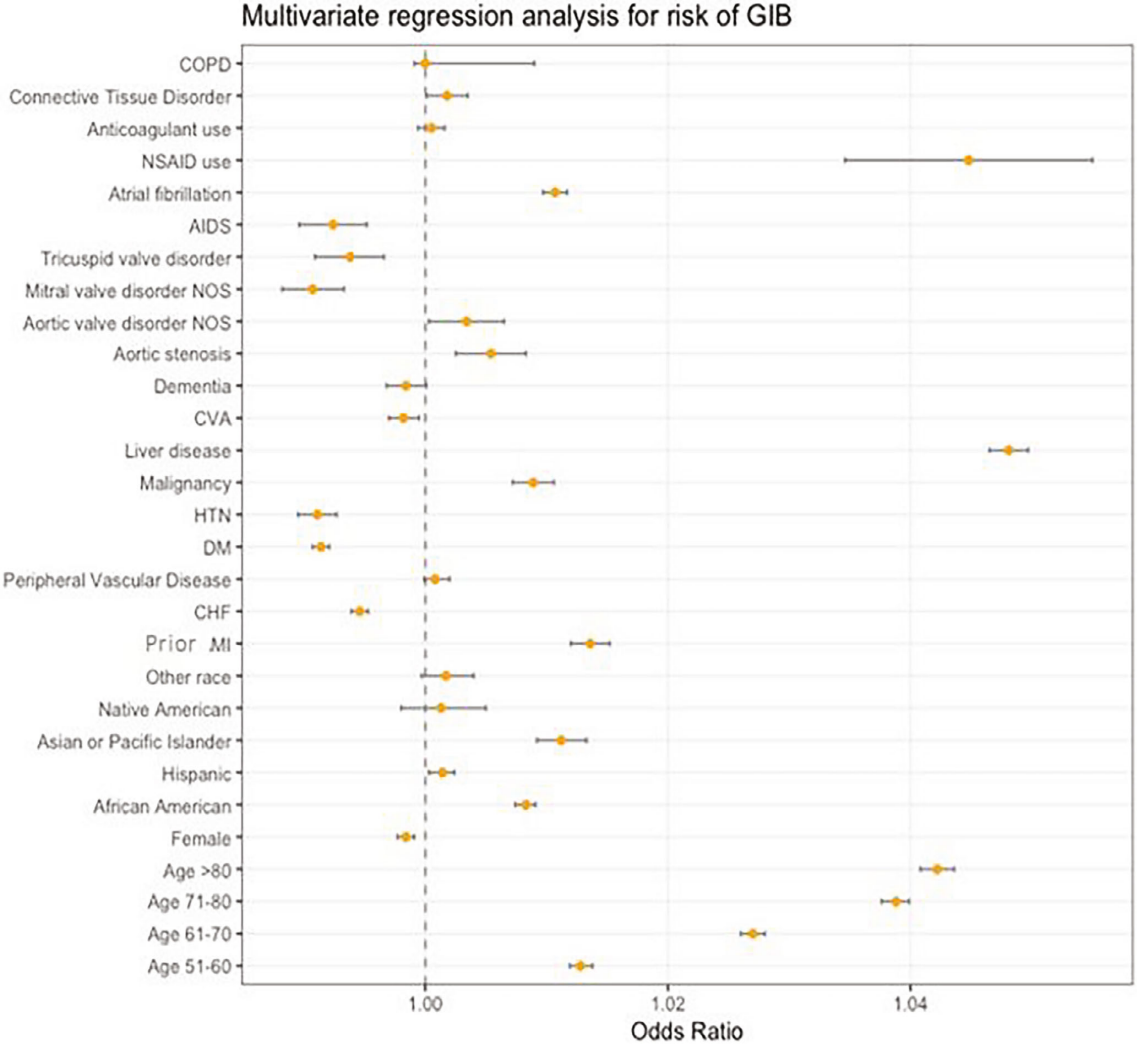
Multivariate regression analysis for risk of GIB. HTN: hypertension; DM: diabetes mellitus; CVA: cerebrovascular disease; CHF: congestive heart failure; MI: myocardial infarction; AIDS: acquired immunodeficiency syndrome; NOS: not otherwise specified; other race: any race other than listed.

In ESRD patients, there was increased risk of GIB with increasing age (Supplementary Table 4). On multivariate analysis (Supplementary Table 4; Figure 2), Asians were found to be at highest risk for GIB when compared with Caucasians (adj.OR = 1.01; 95% CI 1.009–1.013; *p* < 0.01). Patients with prior MI (adj.OR = 1.014; 95% CI 1.012–1.015; *p* < 0.01), malignancy (adj.OR = 1.01; 95% CI 1.007–1.011; *p* < 0.01), liver disease (adj.OR = 1.05; 95% CI 1.04–1.06; *p* < 0.01), atrial fibrillation (adj.OR = 1.011; 95% CI 1.009–1.012; *p* < 0.01), and long-term use of Non-steroidal Anti-inflammatory Drugs (NSAIDs) (adj.OR = 1.045; 95% CI 1.035–1.055; *p* < 0.01) had increased risk of developing GIB (Supplementary Table 4). Among patients with valvular heart diseases, AS was found to be associated with increased risk of GIB (adj.OR = 1.005; 95% CI 1.003–1.008; *p* < 0.01).

Out of 82,330 patients with ESRD and GIB, 6521 had valvular heart disease. Comparison of baseline characteristics between the two groups is summarized in Table 2. Patients with valvular heart disease were older (71.5 ± 12.2 vs. 66.3 ± 13.4; *p* < 0.01). Proportion of female patients was also higher among patients with valvular heart disease when compared with those without valvular disorders (47.5% vs. 44.9%; *p* < 0.01). At baseline, there was no significant difference in mortality (8.6% vs. 9.3%; *p* = 0.05) and length of stay (9.77 ± 13.2 days vs. 9.84 ± 14.7 days; *p* = 0.7) between patients with and without valvular heart disease (Table 3). Propensity score match analysis showed an increased risk for angiodysplasia of colon (OR = 1.02; 95% CI 1.01–1.03; *p* < 0.01) and angiodysplasia of stomach and duodenum (OR = 1.02; 95% CI 1.01–1.03; *p* < 0.01) in patients with valvular heart disease with GIB when compared with patients without valvular heart disease (Table 3). There was no increased risk of mortality (OR = 0.99; 95% CI 0.98–0.995; *p* < 0.01), need for pressors (OR = 0.99; 95% CI 0.98–0.996; *p* < 0.01), or need for mechanical ventilation (OR = 0.98; 95% CI 0.97–0.99; *p* < 0.01) with the presence of valvular disorders (Table 3). However, patients with valvular heart disease had higher need for blood transfusions (OR = 1.06; 95% CI 1.04–1.07; *p* < 0.01).

Among patients with valvular heart disorders and GIB, subgroup analysis was carried out for patients with AS (Tables 4, 5, and 6). Propensity score match analysis showed increased risk of lower GIB (OR = 1.04; 95% CI 1.01–1.06; *p* = 0.02), increased risk of colonic angiodysplasia (OR = 1.03; 95% CI 1.01–1.05; *p* < 0.01), stomach and duodenal angiodysplasia (OR = 1.03; 95% CI 1.02–1.06; *p* < 0.01), and need for blood transfusion (OR = 1.05; 95% CI 1.02–1.09; *p* < 0.01). However, there was no increased risk of mortality (OR = 0.97; 95% CI 0.95–0.99; *p* < 0.01), need for mechanical ventilation (OR = 0.97; 95% CI 0.95–0.99; *p* < 0.01), or pressor requirement (OR = 0.997; 95% CI 0.99–1.01; *p* = 0.57). There was also no significant difference in length of stay between ESRD and GIB patients with and without AS (9.71 ± 13.7 vs. 8.92 ± 12.3; *p* = 0.06).

## 4. Discussion

We investigated whether the concurrence of ESRD and valvular lesions leads to increased risk and mortality from GIB. The following key findings can be drawn from this retrospective study of patients with ESRD. First, patients with ESRD with valvular heart disorders have a higher risk of GIB from angiodysplasias compared with the ones with ESRD only. Moreover, patients with ESRD and AS have higher risk of lower gastrointestinal bleeding (LGIB) and angiodysplasias compared with patients with ESRD only. However, among patients with ESRD and GIB, there is no significant difference in mortality and length of stay between patients with and without valvular heart disease including AS.

The association of AS with increased risk of GIB from angiodysplasias is well established and is termed HS. The prevalence of GIB related to HS has been described as 1–3% of patients with AS [8]. The pathophysiology for increased risk of GIB in both ESRD and AS has been linked to acquired vWF deficiency. The proposed pathophysiology of acquired vWF deficiency in both AS and ESRD provokes increased structural transformation of the vWF from the globular to elongated form and, hence, increased proteolysis by metalloproteinases. The shearing forces also result in increased activation of metalloproteinases as per some experimental studies, thereby increasing the lysis of vWF. This is one proposed mechanism that, even in the presence of normal vWF multimeters, there can be increased risk of bleeding, which is also what our results portrayed [9].

Hence, this can explain the increased risk of bleeding from angiodysplasia of colon (OR = 1.02; 95% CI 1.01–1.03; *p* < 0.01) and angiodysplasia of stomach and duodenum (OR = 1.02; 95% CI 1.01–1.03; *p* < 0.01) when compared with patients without valvular heart disease. Consistent with these results, our study concluded that ESRD patients with valvular heart disorders have a higher risk of GIB from angiodysplasias compared with the ones with ESRD only. Furthermore, patients with ESRD and AS have higher risk of LGIB and angiodysplasias compared with patients with ESRD only. Moreover, patients in our study with valvular heart disease had a higher need for blood transfusions (OR = 1.06; 95% CI 1.04–1.07; *p* < 0.01). However, among patients with ESRD and GIB, there is no significant difference in mortality and length of stay between patients with and without valvular heart disease including AS despite increased risk of GIB.

The etiology of GIB in ESRD patients is multifactorial, including antiplatelets, unfractionated heparin, uremic platelet dysfunction, gastrointestinal angiodysplasia, and erosive ulcers [10]. In patients with CKD, the most common cause of upper gastrointestinal bleed (UGIB) is peptic ulcer disease (PUD). However, the most common cause of LGIB is angioectasias followed by diverticular diseases and ischemic colitis [11]. According to previous studies, ESRD patients on Hemodialysis (HD) have higher incidence of angiodysplasias (19–32%) compared with the general population (5–6%) [11]. The reason for this observed high prevalence of angiodysplasias remains unclear. It is hypothesized that these lesions are not more common, rather more frequently observed as a result of bleeding more in the setting of uremic platelets and vWF deficiency [12].

Prior studies have shown an increased mortality for ESRD patients hospitalized with UGIB, with the first-month mortality rate of 13.7% [13]. Furthermore, in a study by Garlapati et al., patients with ESRD who developed LGIB had 2.5 times higher risk of in-hospital mortality than those without LGIB after propensity matching [2]. Compatible with the above studies, our results portrayed that ESRD with GIB had a higher all-cause mortality compared with the ESRD without GIB (OR = 1.034; 95% CI 1.031–1.036; *p* < 0.01).

A retrospective study analyzing the impact of comorbidities on GIB has shown that advanced age (age >65 years), renal failure, liver disease, and cardiac arrhythmias are highest predictors of GIB [14]. All these parameters are well-recognized risk factors for rebleeding and death and form components of widely accepted and validated risk-stratification scores that have been used in GIB [15]. In congruence with these findings, our study confirmed these predictors of mortality, including advanced age, atrial fibrillation, prior MI, stroke, CHF, liver disease, and malignancy. Other predictors shown in our study included Hispanic race and history of hematemesis. However, in contrast to these earlier publications, Cox analysis for mortality predictors showed statistically significant higher risk of mortality from hematemesis, whereas no increased risk from melena (HR = 1.70, 95% CI 1.45–1.99; *p* < 0.01). The association remains unclear as both melena and hematemesis are signs of UGIB. However, the bleeding from the right side of the colon and small bowel may also represent melena, and it is possible that the presentations from these were gradually progressive, allowing time for work-up and management without abrupt hemodynamic compromise.

Our study had several strengths that portrayed consistent results. The NIS is the largest publicly available inpatient healthcare database designed to produce U.S. regional and national estimates of inpatient utilization, access, cost, quality, and outcomes. The NIS covers more than 97% of the U.S. population, containing data from around 7 million hospital stays yearly. It estimates about 35 million hospitalizations nationally. The use of NIS thus provided a large sample size contributing overall to a high-power study.

Where most of the findings of our study confirmed the findings from prior literature, there were limitations associated, which may explain some of the unexpected results. Despite concluding that ESRD patients with valvular heart disorders have a higher risk of GIB and thus need for transfusion, especially in relation to AS and angioectasia based on the described pathophysiology, increased risk of mortality was not shown. One major limitation that might explain the unchanged mortality was the characteristic of the database used in this study—in the registry, a considerable number of aortic valve disorders were not specified, and hence, they did not contribute to the total pool of AS. Hence, the appropriate number of AS could not be reported that might have led to GIB leading to a significant increase in mortality. More studies with precise data specification are warranted to further explore the effect of ESRD and valvular disorders like AS on mortality secondary to GIB. According to prior data, cardiovascular disease has been implicated as the most common and first cause of death in patients with ESRD on HD [16]. This might be an important contributing factor to the unchanged mortality since the combined effect of ESRD and valvular disorders including AS may have been preceded by other causes of death, such as cardiovascular. A future study to evaluate an age expectancy in patients with valvular heart disease and ESRD needs to be evaluated as the major cause of mortality in patients with ESRD is from high cardiovascular disease like MI.

## 5. Conclusion

In conclusion, our study revealed significantly higher mortality, length of stay, need for mechanical ventilation, and ECMO in ESRD patients with GIB. We also concluded that ESRD patients with valvular heart disorders have a higher risk of GIB from angiodysplasias compared with the ones with ESRD only. Furthermore, patients with ESRD and AS have higher risk of LGIB and angiodysplasias compared with patients with ESRD only. Moreover, patients in our study with valvular heart disease had a higher need for blood transfusions. However, among patients with ESRD with GIB, there is no significant difference in mortality and length of stay between patients with and without valvular heart disease including AS despite increased risk of GIB.

## Figures and Tables

**Table 1:** Comparison of baseline characteristics between patients with and without GIB.

	No. of GIB	GIB	*p*-Value
Total	1,625,122	82,330	
Age (years)	61.9 ± 15.3	66.7 ± 13.4	<0.01
≤50	21.926	11.404	<0.01
51–60	21.114	17.868	<0.01
61–70	25.831	28.872	<0.01
71–80	19.884	26.338	<0.01
>80	11.245	15.518	<0.01
Female	46.534	45.097	<0.01
Male	53.455	54.889	<0.01
Caucasian	41.003	41.963	<0.01
African American	32.583	33.34	<0.01
Hispanic	15.667	13.672	<0.01
Asian or Pacific Islander	3.6902	4.5573	<0.01
Native American	1.1162	0.9571	<0.01
Other race	2.9391	2.7766	<0.01
Prior MI	5.6844	7.5562	<0.01
CHF	42.847	43.315	<0.01
PVD	15.004	16.014	<0.01
CVA	8.0672	8.2218	<0.01
DVT	3.1	3.6	<0.01
PE	2.8	2.6	<0.01
VWD	0.02	0.05	0.5
Dementia	5.8537	7.193	<0.01
CTD	4.1594	3.8771	<0.01
COPD	18.216	20.078	<0.01
PUD	0.5422	13.013	<0.01
Liver disease	9.7438	18.712	<0.01
Liver mild	8.9329	16.646	<0.01
Liver severe	2.7968	9.8895	<0.01
DM	61.064	56.746	<0.01
DM uncomplicated	17.156	17.48	0.02
DM complicated	45.335	40.406	<0.01
Hemiplegia	1.8601	1.9337	0.15
Malignancy	5.5706	7.6861	<0.01
Tumour	3.3121	4.9605	<0.01
Metastatic tumour	1.4427	1.9215	<0.01
Leukemia	0.4678	0.583	<0.01
Lymphoma	1.4829	1.7272	<0.01
AIDS	1.3168	1.1235	<0.01
HTN	94.184	92.593	<0.01
HTN complicated	93.348	91.94	<0.01
HTN uncomplicated	3.9533	2.2847	<0.01
Anticoagulants	12.444	13.668	<0.01
NSAIDs	0.209	0.4154	<0.01
Aspirin	14.719	13.227	<0.01
Atrial fibrillation	21.12	27.921	<0.01
Mitral valve disorder	3.6557	3.5224	0.04
Mitral valve disorder—NOS	1.6185	1.4102	<0.01
Mitral regurgitation	1.8458	1.9106	0.21
Mitral valve prolapse	0.0514	0.0534	0.8
Mitral stenosis	0.1556	0.1555	1
Aortic valve disorders	3.6921	4.6156	<0.01
Aortic valve disorder—NOS	1.4379	1.7685	<0.01
Aortic regurgitation	0.4873	0.5114	0.42
Aortic stenosis	1.7938	2.3722	<0.01
Tricuspid valve disorders	1.4374	1.3592	0.02
Tricuspid valve disorder—NOS	1.1598	1.0737	<0.01
Tricuspid stenosis	0.0043	0.0024	<0.01
Tricuspid regurgitation	0.2735	0.283	0.59
Pulmonary valve disorder	0.1016	0.0972	1
Pulmonary valve disorder—NOS	0.046	0.0401	0.21
Pulmonary stenosis	0.0019	0.0012	<0.01
Pulmonary regurgitation	0.0539	0.0559	0.81
Valvular heart disorders	7.1724	7.9206	<0.01
EGD	2.192	39.948	<0.01
Colonoscopy	0.791	10.83	<0.01
Blood transfusion	12.767	47.024	<0.01
Died	4.687	9.2749	<0.01
Length of stay	6.65 ± 8.95	9.84 ± 14.5	<0.01
Need for vasopressors	1.1725	2.456	<0.01
ECMO requirement	0.033	0.0899	<0.01
Mechanical ventilation	7.6797	13.91	<0.01

Other race: any race other than listed; LDL: low density lipoprotein; MI: myocardial infarction; CHF: congestive heart failure; PVD: peripheral vascular disease; CVA: cerebral vascular disease; DVT: deep venous thrombosis; PE: pulmonary embolism; vWD: von Willebrand disease; CTD: connective tissue disease; COPD: chronic obstructive pulmonary disease; PUD: peptic ulcer disease; DM: diabetes mellitus; AIDS: acquired immunodeficiency syndrome; HTN: hypertension; NOS: not otherwise specified; EGD: esophagogastroduodenoscopy.

**Table 2:** Valvular heart diseases among ESRD patients with GIB.

	No. of valvular disease	Valvular disease	*p*-Value
Total	75,809	6521	
Age (years)	66.3 ± 13.4	71.5 ± 12.2	<0.01
<50	8277	327	<0.01
50–59	12,922	748	<0.01
60–69	21,828	1513	<0.01
70–79	20,236	2053	<0.01
>80	12,546	1880	<0.01
Female	34,028	3100	<0.01
Race			
Caucasian	31,314	3234	<0.01
African American	25,540	1909	<0.01
Hispanic	10,566	690	<0.01
Asian or Pacific Islander	3426	326	<0.01
Native American	756	32	<0.01
Other race	2121	165	<0.01
Death	7078	558	0.04
LOS	9.84 ± 14.7	9.77 ± 13.2	0.7
Need for vasopressors	1845	177	0.16
ECMO	65	<11	0.17
MV	10,615	837	<0.01
Prior MI	5346	875	<0.01
CHF	31,514	4147	<0.01
PVD	11,752	1432	<0.01
CVA	6195	574	0.08
Dementia	5422	500	0.14
CTD	2916	276	0.13
COPD	14,911	1619	<0.01
Liver disease	14,361	1045	<0.01
Hemiplegia	1514	78	<0.01
AIDS	868	57	0.053
UGIB	29,541 (39)	2367 (36.3)	<0.01
LGIB	17,721 (23.4)	1719 (26.4)	<0.01
GIB—NOS	31,602	2711	0.86
Colon angiodysplasia	4855 (6.4)	591 (9.1)	<0.01
Stomach and duodenum angiodysplasia	6935 (9.2)	731 (11.2)	<0.01
Large intestine diverticulosis	5988 (7.9)	567 (8.7)	0.02
Small intestine diverticulosis	89	<11	<0.01
Esophageal varices and ulcer	3934 (5.2)	203 (3.1)	<0.01
Anticoagulation use	10,198	1055	<0.01
NSAID use	319	23	0.41
Aspirin	9906	984	<0.01
Atrial fibrillation	20,284	2703	<0.01
Hematemesis	1164	62	<0.01
Melena	2364	216	0.39
Colonoscopy	8223	693	0.59
EGD	30,281	2608	0.94
Blood transfusion	35,412	3303	<0.01
DM	43,223	3496	<0.01
HTN	70,126	6106	<0.01
Malignancy	5865	463	0.07

Other race: any race other than listed; LDL: low density lipoprotein; MI: myocardial infarction; CHF: congestive heart failure; PVD: peripheral vascular disease; CVA: cerebral vascular disease; DVT: deep venous thrombosis; PE: pulmonary embolism; vWD: von Willebrand disease; CTD: connective tissue disease; COPD: chronic obstructive pulmonary disease; PUD: peptic ulcer disease; DM: diabetes mellitus; AIDS: acquired immunodeficiency syndrome; HTN: hypertension; NOS: not otherwise specified; GIB: gastrointestinal bleed; UGIB: upper gastrointestinal bleed; EGD: esophagogastroduodenoscopy; LOS: length of stay; LGIB: lower gastrointestinal bleeding; ECMO: extracorporeal membrane oxygenation; MV: mechanical ventilation.

**Table 3:** Propensity match in GIB patients with and without valvular heart disorders.

	OR	95% CI	*p*-Value
Mortality	1.1	1.09–1.11	<0.01
Blood transfusion	1.06	1.03–1.07	<0.01
UGIB	0.99	0.97–1.01	0.26
LGIB	1.01	0.99–1.02	0.53
Angiodysplasia colon	1.02	1.01–1.03	<0.01
Stomach and duodenum angiodysplasia	1.02	1.01–1.03	<0.01
Mechanical ventilation	0.98	0.97–0.99	<0.01
Need for vasopressors	0.99	0.98–0.99	<0.01

UGIB: upper GI bleed; LGIB: lower GI bleed.

**Table 4:** Propensity match in GIB with and without aortic valve disorder.

	OR	95% CI	*p*-Value
Mortality	0.998	0.985–1.011	0.71
Blood transfusion	1.07	1.05–1.1	<0.01
UGIB	0.98	0.96–1.01	0.13
LGIB	1.017	0.997–1.034	0.1
Angiodysplasia colon	1.02	1.01–1.04	<0.01
Stomach and duodenum angiodysplasia	1.02	1.01–1.04	<0.01

UGIB: upper GI bleed; LGIB: lower GI bleed.

**Table 5:** Propensity matching in GIB with and without aortic stenosis.

	OR	95% CI	*p*-Value
Mortality	0.97	0.95–0.99	<0.01
Blood transfusion	1.05	1.02–1.09	<0.01
UGIB	0.99	0.95–1.02	0.34
LGIB	1.03	1.01–1.06	0.02
Angiodysplasia colon	1.03	1.01–1.05	<0.01
Stomach and duodenum angiodysplasia	1.04	1.02–1.06	<0.01
Mechanical ventilation	0.97	0.95–0.99	<0.01
Need for vasopressors	0.99	0.99–1.01	0.57

UGIB: upper GI bleed; LGIB: lower GI bleed.

**Table 6:** Comparison of baseline characteristics in patients with aortic stenosis with and without GIB.

	No. of GIB	GIB
Total	29,152	1953
Age (years)	72.4 ± 11.6	74.2 ± 10.5
<50	1040	37
50–59	3029	140
60–69	6775	441
70–79	9399	654
>80	8909	681
Caucasian	17,134	1088
African American	6102	467
Hispanic	3217	223
Asian or Pacific Islander	1058	84
Native American	152	<11
Other race	742	46
Female	12,700	889
LOS	6.94 ± 7.77	8.96 ± 12.3
Need for vasopressors	605	54
ECMO	27	<11
MV	2266	192
Prior MI	3739	292
CHF	20,065	1234
PVD	6152	399
CVA	2780	176
Dementia	2438	157
CTD	1153	77
COPD	7472	495
Hemiplegia	494	26
AIDS	141	<11
Anticoagulant use	5770	354
NSAID use	60	<11
Atrial fibrillation	11,698	844
Hematemesis		24
Melena		88
Colonoscopy	160	135
EGD	462	671
Blood transfusion	3506	898
Liver disease	2525	296
Malignancy	1812	161
DM	17,690	1113
HTN	27,918	1848
UGIB		726
LGIB		555
GIB NOS		715
Angiodysplasia colon		209
Stomach and duodenum angiodysplasia		256
Diverticulosis small intestine		<11
Diverticulosis large intestine		196
Esophageal varices and ulcer		55
Gastritis		183
Peptic ulcer		16
Gastric ulcer		145
Duodenal ulcer		125
Gastrojejunal ulcer		<11
Anorectal hemorrhoid		150
Dieulafoy lesion stomach		16
Dieulafoy lesion intestine		13

Other race: any race other than listed; LDL: low density lipoprotein; MI: myocardial infarction; CHF: congestive heart failure; PVD: peripheral vascular disease; CVA: cerebral vascular disease; DVT: deep venous thrombosis; PE: pulmonary embolism; vWD: von Willebrand disease; CTD: connective tissue disease; COPD: chronic obstructive pulmonary disease; PUD: peptic ulcer disease; DM: diabetes mellitus; AIDS: acquired immunodeficiency syndrome; HTN: hypertension; NOS: not otherwise specified; GIB: gastrointestinal bleed; UGIB: upper gastrointestinal bleed; EGD: esophagogastroduodenoscopy; LOS: length of stay; LGIB: lower gastrointestinal bleeding; ECMO: extracorporeal membrane oxygenation; MV: mechanical ventilation.

## Data Availability

The data used to support the findings of this study are included within the article.

## Supplementary Materials

Supplementary Materials Contains supplementary Tables 1–4 referenced in the manuscript text.
