# Supplementary material for: Risk of Gastrointestinal Bleeding in Patients with End-Stage Renal Disease: The Link between Gut, Heart, and Kidneys
Source: Gastroenterol Res Pract. 2023 May 8;2023:9986157. doi: 10.1155/2023/9986157 (PMC10185431; doi:10.1155/2023/9986157)
Supplement: Supplementary Materials — Contains supplementary Tables 1–4 referenced in the manuscript text. [file 9986157.f1.docx]

**Supplementary File**

**Mortality Analysis (in GIB population)**

**Supplementary Table 2: Univariate mortality in GIB:**

|  | **OR** | **95% CI** | **P value** |
| --- | --- | --- | --- |
| Age | 1.0007 | 1.0006-1.0009 | <0.01 |
| <50 | Reference | | |
| 50-60 | 1.01 | 1-1.014 | 0.09 |
| 60-70 | 1.012 | 1.005-1.019 | <0.01 |
| 70-80 | 1.015 | 1.008-1.022 | <0.01 |
| >80 | 1.033 | 1.025-1.041 | <0.01 |
| Caucasian | Reference | | |
| African American | 0.975 | 0.97-0.979 | <0.01 |
| Hispanic | 0.996 | 0.989-1.001 | 0.17 |
| Asian or Pacific Islander | 1.001 | 0.99-1.011 | 0.89 |
| Native American | 0.996 | 0.974-1.019 | 0.75 |
| Other | 1.019 | 1.005-1.033 | <0.01 |
| Female | 0.993 | 0.989-0.997 | <0.01 |
| AMI | 1.13 | 1.12-1.14 | <0.01 |
| CHF | 1.026 | 1.02-1.03 | <0.01 |
| PVD | 1.002 | 0.997-1.01 | 0.49 |
| CVA | 1.040 | 1.03-1.05 | <0.01 |
| Dementia | 1.01 | 1.001-1.02 | 0.02 |
| CTD | 0.995 | 0.985-1.005 | 0.3 |
| COPD | 0.998 | 0.993-1.003 | 0.39 |
| Hemiplegia | 1.09 | 1.07-1.11 | <0.01 |
| AIDS | 0.99 | 0.97-1.01 | 0.37 |
| Atrial fibrillation | 1.031 | 1.026-1.036 | <0.01 |
| Hematemesis | 1.057 | 1.037-1.079 | <0.01 |
| Melena | 0.97 | 0.96-0.98 | <0.01 |
| DM | 0.964 | 0.96-0.968 | <0.01 |
| HTN | 0.92 | 0.907-0.925 | <0.01 |
| Liver disease | 1.059 | 1.053-1.065 | <0.01 |
| Malignancy | 1.053 | 1.044-1.06 | <0.01 |
| Mitral stenosis | 0.991 | 0.95-1.04 | 0.78 |
| Mitral regurgitation | 0.9879 | 0.9744-1.0016 | 0.08 |
| Mitral valve disorder NOS | 0.9967 | 0.9804-1.0134 | 0.7 |
| Aortic stenosis | 0.9894 | 0.9773-1.0017 | 0.09 |
| Aortic regurgitation | 0.98 | 0.9568-1.0056 | 0.13 |
| Aortic valve disorder NOS | 1.004 | 0.989-1.02 | 0.6 |
| Tricuspid regurgitation | 1.105 | 0.98-1.06 | 0.47 |
| Tricuspid valve disorder NOS | 0.998 | 0.979-1.017 | 0.81 |
| Pulmonary regurgitation | 0.97 | 0.91-1.05 | 0.45 |
| Pulmonary valve disorder NOS | 0.939 | 0.886-0.996 | 0.04 |
| **Multivariate mortality in GIB** | | | |

| Age |  |  |  |
| --- | --- | --- | --- |
| <50 | Reference | | |
| 50-60 | 1.007 | 1.0006058-1.014061 | 0.03 |
| 60-70 | 1.01546 | 1.0090928-1.021871 | <0.01 |
| 70-80 | 1.02819 | 1.0215893-1.034845 | <0.01 |
| >80 | 1.0491 | 1.0413482-1.056923 | <0.01 |
| Caucasian | Reference | | |
| African American | 0.9867 | 0.9826572-0.990880 | <0.01 |
| Hispanic | 1.0067 | 1.0002-1.0133 | 0.043 |
| Asian or Pacific Islander | 1.000179 | 0.9909024-1.009543 | 0.97 |
| Native American | 1.00576 | 0.9860948-1.025823 | 0.57 |
| Other race | 1.0198 | 1.0062-1.0336 | <0.01 |
| Female | 1.002134 | 0.9984892-1.005792 | 0.25 |
| Prior MI | 1.13 | 1.12-1.14 | <0.01 |
| CHF | 1.022 | 1.018-1.026 | <0.01 |
| CVA | 1.039 | 1.03-1.047 | <0.01 |
| Dementia | 1.007 | 0.999-1.015 | 0.1 |
| Hemiplegia | 1.073 | 1.054-1.093 | <0.01 |
| Atrial Fibrillation | 1.023 | 1.018-1.028 | <0.01 |
| Hematemesis | 1.06 | 1.04-1.081 | <0.01 |
| Melena | 0.965 | 0.956-0.975 | <0.01 |
| DM | 0.969 | 0.965-0.973 | <0.01 |
| HTN | 0.926 | 0.917-0.935 | <0.01 |
| Liver disease | 1.062 | 1.055-1.068 | <0.01 |
| Malignancy | 1.051 | 1.042-1.061 | <0.01 |
| MR | 0.976 | 0.952-0.991 | <0.01 |
| AS | 0.973 | 0.961-0.985 | <0.01 |
| AR | 0.987 | 0.961-1.014 | 0.34 |
| Pulmonary valve disorder | 0.946 | 0.895-1 | 0.05 |

Other Race: Any race other than listed ;LDL: Low Density Lipoprotein; MI: Myocardial Infarction; CHF: Congestive Heart Failure; PVD: Peripheral Vascular Disease; CVA: Cerebral Vascular Disease ; DVT: Deep Venous Thrombosis; PE: Pulmonary Embolism; VWD: Von Willebrand Disease; CTD: Connective Tissue Disease; COPD: Chronic Obstructive Pulmonary Disease; PUD: Peptic Ulcer Disease; DM: Diabetes Mellitus; AIDS: Acquired Immunodeficiency Syndrome; HTN: Hypertension; NOS: Not Otherwise Specified; EGD: Esophagogastroduodenoscopy; MS: Mitral Stenosis; AS: Aortic Stenosis; AR: Aortic Regurgitation

**Mortality Analysis (in GIB population)**

**Supplementary Table 3: Cox analysis for mortality in GIB- censored at LOS:**

|  | **HR** | **95% CI** | | | **p value** |
| --- | --- | --- | --- | --- | --- |
|  |  | Lower | Upper | |  |
| Age <50 years | Reference | | | | |
| 51-60 years | 1.142461 | 1.03412845 | | 1.2621416 | <0.01 |
| 61-70 years | 1.276548 | 1.163445 | | 1.4006465 | <0.01 |
| 71-80 years | 1.483099 | 1.3476686 | | 1.6321386 | <0.01 |
| >80 years | 1.9798 | 1.78800297 | | 2.1921712 | <0.01 |
| Female | 1.005651 | 0.95945908 | | 1.054066 | 0.81 |
| Caucasian | Reference | | | | |
| African American | 0.873241 | 0.82506942 | | 0.9242255 | <0.01 |
| Hispanic | 1.047965 | 0.97672539 | | 1.124401 | 0.19 |
| Asian or Pacific Islander | 0.970817 | 0.86898793 | | 1.0845783 | 0.6 |
| Native American | 1.240871 | 0.98347391 | | 1.5656355 | 0.07 |
| Other race | 1.002426 | 0.88312607 | | 1.1378418 | 0.97 |
| Prior MI | 1.60982 | 1.50903838 | | 1.7173332 | <0.01 |
| CHF | 1.019854 | 0.97122811 | | 1.0709139 | 0.43 |
| CVA | 1.033555 | 0.95876436 | | 1.1141791 | 0.39 |
| Dementia | 1.075014 | 0.98576921 | | 1.1723383 | 0.1 |
| Hemiplegia | 1.009351 | 0.89097345 | | 1.1434562 | 0.88 |
| Atrial fibrillation | 1.1745 | 1.11504163 | | 1.2371297 | <0.01 |
| Hematemesis | 1.702847 | 1.45685144 | | 1.9903796 | <0.01 |
| Melena | 0.680817 | 0.58236984 | | 0.7959054 | <0.01 |
| DM | 0.919769 | 0.87711199 | | 0.9644996 | <0.01 |
| HTN | 0.709149 | 0.65840503 | | 0.7638034 | <0.01 |
| Liver disease | 1.642459 | 1.55587504 | | 1.7338623 | <0.01 |
| Malignancy | 1.329457 | 1.2355436 | | 1.4305093 | <0.01 |
| MR | 0.851029 | 0.70901092 | | 1.0214946 | 0.08 |
| AS | 0.877943 | 0.74804772 | | 1.0303951 | 0.11 |
| AR | 0.920622 | 0.63811301 | | 1.3282049 | 0.66 |
| Pulmonary valve disorder- NOS | 0.291842 | 0.03765247 | | 2.2620436 | 0.24 |

 Other Race: Any race other than listed; LDL: Low Density Lipoprotein; MI: Myocardial Infarction; CHF: Congestive Heart Failure; PVD: Peripheral Vascular Disease; CVA: Cerebral Vascular Disease ; DVT: Deep Venous Thrombosis; PE: Pulmonary Embolism; VWD: Von Willebrand Disease; CTD: Connective Tissue Disease; COPD: Chronic Obstructive Pulmonary Disease; PUD: Peptic Ulcer Disease; DM: Diabetes Mellitus; AIDS: Acquired Immunodeficiency Syndrome; HTN: Hypertension; NOS: Not Otherwise Specified; EGD: Esophagogastroduodenoscopy; MS: Mitral Stenosis; AS: Aortic Stenosis; AR: Aortic Regurgitation

**Risk of GIB**

**Supplementary Table 4: Univariate analysis for risk of GIB**

|  | **OR** | **95%CI** | **P value** |
| --- | --- | --- | --- |
| Age <50 | Reference | | |
| 50-59 | 1.014656 | 1.013764-1.015549 | <0.01 |
| 60-69 | 1.027938 | 1.027034-1.028844 | <0.01 |
| 70-79 | 1.037815 | 1.036778-1.038852 | <0.01 |
| >80 | 1.040983 | 1.039724-1.042243 | <0.01 |
| Caucasian | Reference | | |
| African American | 0.999 | 0.9991696-1.000816 | 0.99 |
| Hispanic | 0.993 | 0.9920142-0.994130 | <0.01 |
| Asian or Pacific Islander | 1.01 | 1.0075409-1.011735 | <0.01 |
| Native American | 0.992 | 0.9891625-0.995587 | <0.01 |
| Other race | 0.996 | 0.9943466-0.998437 | <0.01 |
| Female | 0.997 | 0.9967-0.998 | <0.01 |
| AMI | 1.016 | 1.014-1.018 | <0.01 |
| CHF | 1.001 | 1.0002-1.0016 | 0.013 |
| PVD | 1.004 | 1.003-1.005 | <0.01 |
| CVA | 1.001 | 0.99-1.002 | 0.126 |
| Dementia | 1.011 | 1.01-1.013 | <0.01 |
| Hemiplegia | 1.002 | 0.99-1.004 | 0.143 |
| PUD | 1.66 | 1.65-1.67 | <0.01 |
| DM | 0.992 | 0.991-0.992 | <0.01 |
| HTN | 0.987 | 0.985-0.989 | <0.01 |
| Malignancy | 1.018 | 1.017-1.02 | <0.01 |
| Liver disease | 1.046 | 1.045-1.048 | <0.01 |
| Valvular heart disease | 1.005 | 1.004-1.006 | <0.01 |
| Aortic stenosis | 1.015 | 1.012-1.018 | <0.01 |
| Aortic regurgitation | 1.002 | 0.998-1.007 | 0.35 |
| Aortic NOS | 1.011 | 1.01-1.014 | <0.01 |
| Mitral regurgitation | 1.002 | 0.999-1.004 | 0.19 |
| Mitral stenosis | 1 | 0.992-1.008 | 0.99 |
| Mitral NOS | 0.994 | 0.992-0.996 | <0.01 |
| Tricuspid | 0.997 | 0.995-1 | 0.057 |
| Pulmonary | 0.998 | 0.988-1.01 | 0.7 |
| AIDS | 0.993 | 0.991-0.996 | <0.01 |
| Atrial fibrillation | 1.019 | 1.018-1.02 | <0.01 |
| NSAIDS | 1.044 | 1.034-1.054 | <0.01 |
| Anticoagulation use | 1.005 | 1.004-1.006 | <0.01 |
| CTD | 0.997 | 0.995-0.998 | <0.01 |
| COPD | 1.006 | 1.005-1.007 | <0.01 |
| Multivariate Analysis For Risk of GI Bleed | | | |

|  | **HR** | **95% CI** | **p-value** |
| --- | --- | --- | --- |
| Age <50 years | Reference | | |
| 51-60 years | 1.0128 | 1.0119-1.0138 | <0.01 |
| 61-70 years | 1.027 | 1.026-1.028 | <0.01 |
| 71-80 years | 1.0388 | 1.0376-1.0399 | <0.01 |
| >80 years | 1.0422 | 1.0408-1.0436 | <0.01 |
| Female | 0.9984 | 0.9977-0.9991 | <0.01 |
| Caucasian | Reference | | |
| African American | 1.0083 | 1.0074-1.0091 | <0.01 |
| Hispanic | 1.0014 | 1.0003-1.0024 | 0.01 |
| Asian or Pacific Islander | 1.0112 | 1.0092-1.0133 | <0.01 |
| Native American | 1.0013 | 0.998-1.005 | 0.44 |
| Other race | 1.0017 | 0.9997-1.004 | 0.1 |
| Prior MI | 1.0136 | 1.012-1.0152 | <0.01 |
| CHF | 0.9946 | 0.9939-0.9953 | <0.01 |
| PVD | 1.0008 | 0.9999-1.002 | 0.09 |
| DM | 0.9914 | 0.9907-0.9921 | <0.01 |
| HTN | 0.9911 | 0.9895-0.9927 | <0.01 |
| Malignancy | 1.0089 | 1.0072-1.0106 | <0.01 |
| Liver disease | 1.0481 | 1.0465-1.0497 | <0.01 |
| CVA | 0.9982 | 0.997-0.9995 | 0.01 |
| Dementia | 0.9984 | 0.9968-1.0001 | 0.06 |
| AS | 1.0054 | 1.0025-1.0083 | <0.01 |
| Aortic Valve Disorder - NOS | 1.0034 | 1.0003-1.0065 | 0.03 |
| Mitral valve disorder-NOS | 0.9907 | 0.9882-0.9933 | <0.01 |
| Tricuspid valve disorder | 0.9938 | 0.9909-0.9966 | <0.01 |
| AIDS | 0.9924 | 0.9896-0.9952 | <0.01 |
| Atrial fibrillation | 1.0107 | 1.0097-1.0117 | <0.01 |
| NSAID use | 1.0448 | 1.0346-1.055 | <0.01 |
| Anticoagulation use | 1.0005 | 0.9994-1.0016 | 0.36 |
| CTD | 1.0018 | 1.0001-1.0035 | 0.04 |
| COPD | 1 | 0.9991-1.009 | 1 |

 Other Race: Any race other than listed; LDL: Low Density Lipoprotein; MI: Myocardial Infarction; CHF: Congestive Heart Failure; PVD: Peripheral Vascular Disease; CVA: Cerebral Vascular Disease ; DVT: Deep Venous Thrombosis; PE: Pulmonary Embolism; VWD: Von Willebrand Disease; CTD: Connective Tissue Disease; COPD: Chronic Obstructive Pulmonary Disease; PUD: Peptic Ulcer Disease; DM: Diabetes Mellitus; AIDS: Acquired Immunodeficiency Syndrome; HTN: Hypertension; NOS: Not Otherwise Specified

**Supplementary Table 7: Propensity match GIB and no GIB (ESRD population):**

|  | OR | 95% CI | P-value |
| --- | --- | --- | --- |
| Mortality | 1.034 | 1.031-1.037 | <0.01 |
| Blood transfusion | 1.433 | 1.42-1.44 | <0.01 |
| Mechanical ventilation | 1.07 | 1.065-1.073 | <0.01 |
| Need for Vasopressors | 1.0005 | 1.0002-1.0006 | <0.01 |
